# Impact of Vessel Size, Dose Levels, and Body Habitus on Iodine Quantification in Cardiovascular Photon-Counting Computed Tomography

**DOI:** 10.1093/bjr/tqaf181

**Published:** 2025-11-01

**Authors:** Martin V. Rybertt, Leening P. Liu, Manoj Mathew, Pooyan Sahbaee, Harold I. Litt, Peter B. Noël

**Affiliations:** 1 Department of Radiology, Perelman School of Medicine, University of Pennsylvania, Philadelphia, PA, 19104, USA; 2 Department of Bioengineering, University of Pennsylvania, Philadelphia, PA, 19104, USA; 3 Siemens Healthineers, Malvern, PA, 19355, USA

## Abstract

**Objectives:**

This study evaluates the performance of a clinical dual-source photon-counting computed tomography (PCCT) system in quantifying iodine within calcified vessels, using 3D-printed phantoms with vascular-like structures lined with calcium.

**Methods:**

Parameters assessed include lumen diameters (4, 6, 8, 10, and 12 mm), phantom sizes (S: 20×20 cm, M: 25×25 cm, L: 30×40 cm), and iodine concentrations (2, 5, and 10 mg/mL). Scans were performed with a cardiac high-pitch acquisition protocol at radiation dose levels of 5 and 10 mGy to systematically evaluate iodine quantification accuracy and spectral imaging performance.

**Results:**

The results indicate that for lumen diameters ≥6 mm, iodine quantification remains stable across all dose levels and smaller phantom sizes, where error remained consistently below 0.9 mg/mL. Furthermore, iodine quantification revealed a significant dependence on phantom size while selected radiation dose levels were insignificant. Virtual Monoenergetic Imaging (VMI) at 70 keV showed stable performance for larger lumens (≥6 mm) with variations of 20.3 ± 13.2 HU across all conditions, while smaller lumens remained stable in medium to small phantoms.

**Conclusions:**

These findings highlight the influence of lumen diameter, patient size, and radiation dose in optimizing PCCT protocols for spectral imaging. Results indicate that PCCT maintains stable and precise imaging performance across diverse patient anatomies, with robust differentiation of iodine and calcium in adjacent regions.

**Advances in knowledge:**

This study demonstrates PCCT's potential to enhance spectral imaging in vascular applications, characterizing iodine quantification at relevant lesion sizes for vascular imaging.

## Introduction

Coronary Artery Disease (CAD) is the leading cause of death in the United States, with over 300,000 fatalities reported in 2023^1^. This high mortality rate underscores the critical need for effective diagnostic tools and early detection methods. Currently, one of the primary diagnostic tools for CAD is computed tomography (CT), which has been associated with a reduction in cardiovascular mortality^2^. Diagnosing CAD presents significant challenges due to the small diameters of coronary vessels and the difficulty in differentiating between calcified plaque and iodinated contrast^3–5^. Traditional CT imaging may not provide the necessary contrast resolution to distinguish these accurately, leading to potential diagnostic ambiguities. Spectral CT addresses this issue by leveraging the attenuation characteristics of different materials to produce material-specific reconstructions^6,7^. For example, Spectral CT can generate maps that isolate iodine, thereby enhancing the visibility of contrast agents used during imaging. However, despite these advances, there are still challenges to overcome and differentiating between materials with similar attenuation profiles, such as calcium and iodine, remains a significant obstacle^8^.

The clinically available dual-source PCCT represents an advance over other Spectral CT technology by offering improved material decomposition, reduced radiation dose, and enhanced spatial resolution, making it particularly well-suited for cardiovascular applications^9–11^. For diagnosing CAD, this scanner uniquely combines these improvements with the temporal benefits of dual-source imaging^12^. Studies have already explored a clinical prototype PCCT's applications for CAD diagnosis showing that it provides greater image quality and diagnostic confidence than a dual-layer Spectral CT^13^. Additionally, PCCT shows improved lesion detectability compared to conventional CT, especially in larger patients^14^. PCCT was also used to assess stent and plaque composition^15,16^, demonstrating its ability to reduce blooming artifacts and provide clearer views of stent structures^17,18^. Additionally, PCCT has been evaluated for calcium scoring directly from contrast enhanced scans, thus reducing radiation exposure while achieving performance comparable to non-contrast CT^19–22^. While PCCT has demonstrated strong diagnostic capabilities, with multiple studies highlighting its advantages in technical performance evaluations^23,24^, a critical question for CAD diagnostics remains: can PCCT accurately quantify iodine in relation to vessel diameter and calcium presence. The spatial resolution in the spectral mode of the clinically approved PCCT provides high-quality spectral data at superior resolution than conventional CT. Although iodine quantification may be secondary in CAD diagnostics, its reliable association with virtual non-contrast imaging is crucial for occlusion detection. The performance of virtual non-contrast imaging directly depends on accurate iodine quantification, as iodine removal requires precise determination of its concentration.

This study aims to evaluate how target (lumen) size, patient habitus, iodine concentration, and radiation dose collectively influence the accuracy of spectral imaging, with an emphasis on iodine quantification. By systematically analyzing how these factors interact, we seek to better understand their combined impact on iodine measurement reliability, particularly for potential applications for CAD diagnostics.

## Methods

### Phantom Design.

A set of 3-D printed phantoms were designed for use as inserts for a commercially available technical phantom (Multi-energy CT Phantom, Sun Nuclear, Melbourne, FL, USA) to evaluate the performance of iodine quantification in small calcified coronary vessels. All phantoms were printed (TAZ Sidekick, Lulzbot, Fargo, ND, USA) using a calcium-doped polylactic acid (PLA) filament (StoneFil, FormFutura, AM Nijmegen, The Netherlands) to mimic coronary calcifications. Each 3-D printed insert contains a constant diameter u-shaped tube that can be filled with iodinated contrast. Moreover, each tube has a lumen diameter ranging from 4 to 12 mm (4, 6, 8, 10, 12 mm) and is surrounded by a calcium lining of 1 mm with a Hounsfield unit of 380±24 HU to imitate the anatomy of coronary vessels with advanced atherosclerosis.

### Image Acquisition.

**Figure 1.**
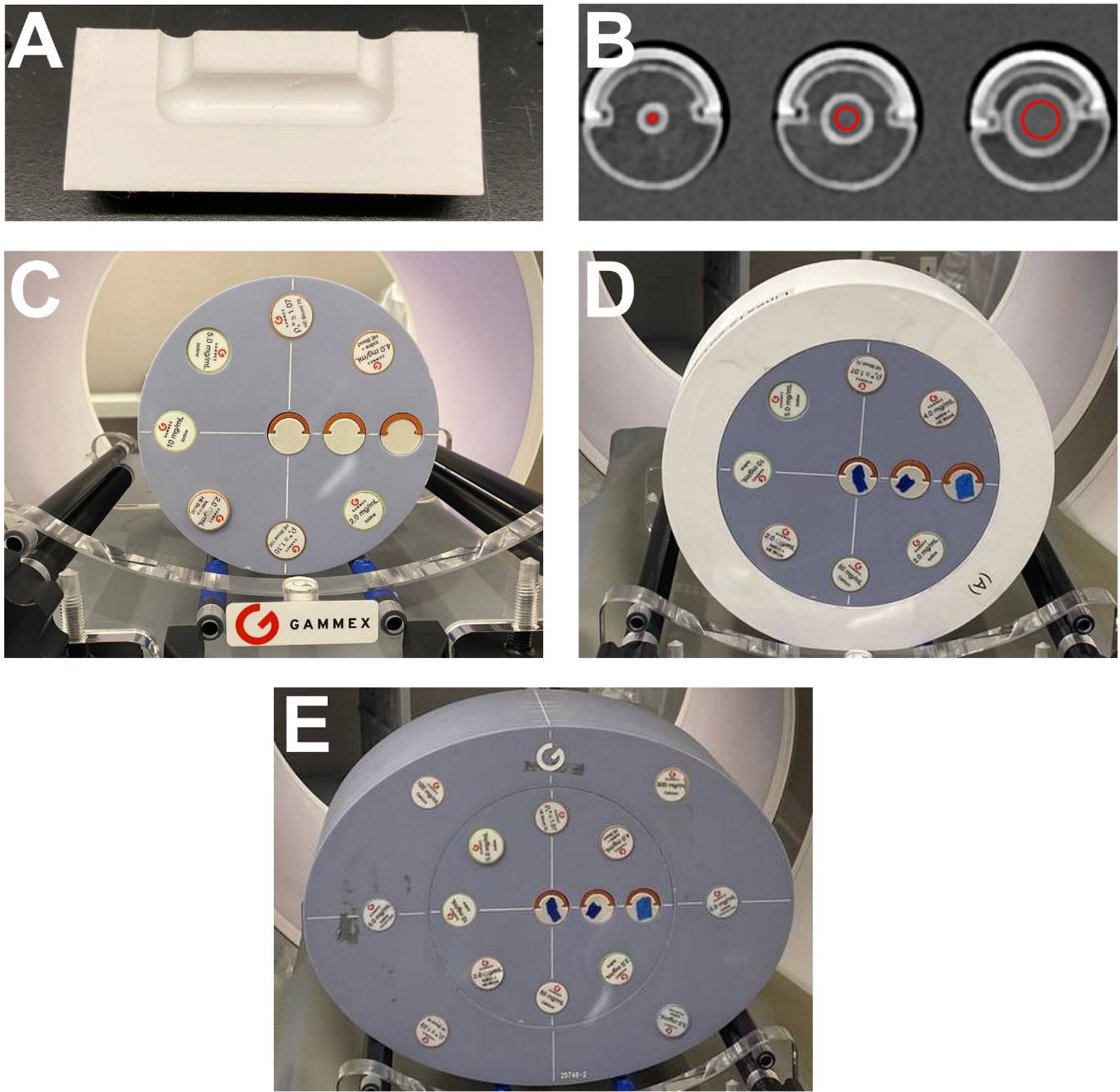
Experimental setup on PCCT scanner. Phantom rod inserts consist of a constant diameter u-shaped tube that can be filled with contrast material (A) with each insert having a different diameter and a 1 mm calcium lining (B). Rods were scanned in three separate simulated patient sizes: small (C), medium (D), and large (E).

The 3-D printed phantoms were scanned on a first-generation clinical dual-source PCCT (NAEOTOM Alpha, Siemens Healthineers, Munich, Germany) within the technical phantom consisting of various tissue-mimicking and material-specific inserts and an outer extension ring. To determine how patient habitus impacts iodine quantification, the 3-D printed inserts were scanned in different sized phantoms. The smallest setup (S) used the inner part of the technical phantom with a diameter of 20 cm. For the medium (M) setting, the S phantom was positioned within a 3-D printed PLA ring, increasing the diameter to 25 cm. The large (L) setting was achieved by placing the inner phantom inside the 30×40 cm extension ring of the technical phantom (Figure 1).

After filling the inserts with a 2 mg/mL iodine solution (Isovue-300, Bracco Diagnostics, Milan, Italy) and positioning them within each phantom configuration, scans were conducted at a tube voltage of 120 kVp using a high-pitch Flash acquisition with a pitch of 3.2 and rotation time of 0.25 s across two volumetric CT dose index (CTDI_vol_) levels (5, and 10 mGy), a ECG monitor was simulated at 60 bpm, and the scan triggered at 70% of the heart cycle (Table 1). Each dose level was repeated three times to evaluate reproducibility. Subsequently, this procedure was repeated for higher iodine concentrations (5 and 10 mg/mL) to assess the effects of varying contrast levels on quantification accuracy.

### Image Reconstruction.

Spectral results were reconstructed for each scan, including virtual monoenergetic images (VMI) at 70 keV, iodine density (ID), and virtual non contrast (VNC). After expert review by a board-certified cardiothoracic radiologist (HL), image reconstruction was implemented using departmental parameters: a field of view of 180 mm, slice thickness and increment of 0.4 mm, a Bv40 filter, and Quantum Iterative Reconstruction at level 3.

### Image Analysis.

Regions of interest (ROI) of 60% of tube diameter were drawn manually for each insert using the VMI 70 keV images from the 10 mGy scans for each phantom size. ROI placement details, including coordinates and size, were recorded and reused for measuring additional spectral parameters within the same phantom size. The mean of each ROI was determined, and the standard deviation across slices was calculated for twenty consecutive central slices of three scans (60 slices total), while the standard deviation within a singular ROI was recorded as noise.

To illustrate the effects of phantom size and lumen diameter on image quality, VMI 70 keV slices were visually compared in the coronal plane and verified by a board-certified radiologist. Scatter plots were generated to visualize the influence of lesion size, phantom size, iodine concentration, and dose for iodine density, VMI, and VNC images with each point representing mean measurements from ROIs and their corresponding standard deviations. Finally, to quantify deviations in lumen diameter, we calculated the relative difference from a reference value, defined as the diameter of the 12 mm insert within the small phantom at a dose of 10 mGy for each spectral result (ID, VMI, and VNC).

### Statistical Analysis.

Samples were divided by spectral result and tested for normality using a Shapiro-Wilk test. In parallel, each sample was evaluated for homoscedasticity via the Levene test, where a p-value under 0.05 was considered significant. As variance equality could not be assumed, the Kruskal–Wallis non-parametric test was applied to examine differences among groups defined by lumen diameter, phantom size, iodine concentration, and dose level. In the case of lumen diameter and phantom size, Dunn post-hoc tests were applied to determine statistical differences between specific parameters. Because of the multiple comparison tests performed (n = 288), a Bonferroni correction was performed resulting in a significance threshold of 1.736 × 10^−4^. All statistical analysis was implemented on Python.

## Results

**Figure 2.**
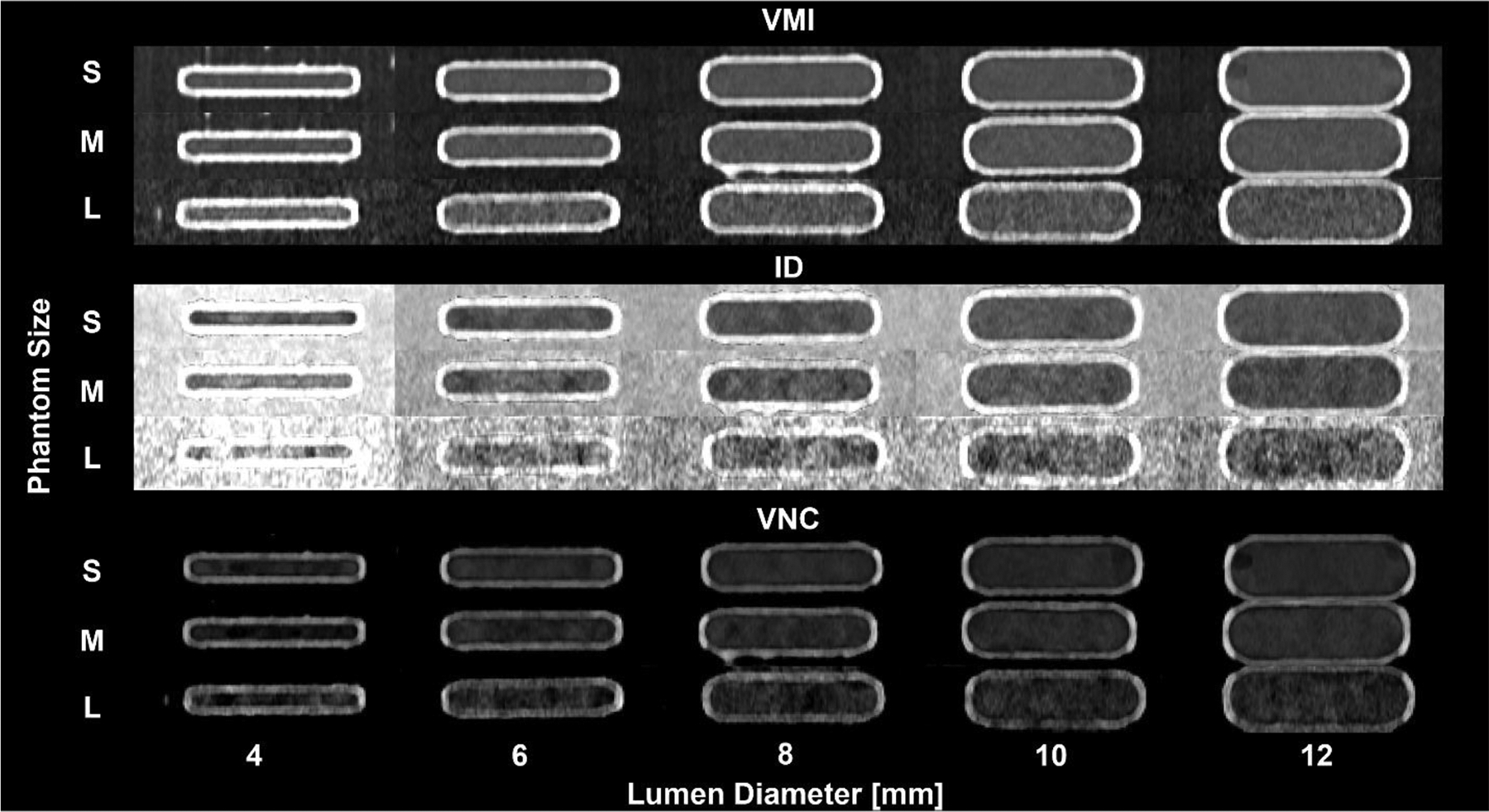
Coronal images of spectral results with varying phantom sizes and lumen diameter. 3-D printed phantoms were filled with a solution with an iodine concentration of 5 mg/mL at a radiation dose of 10 mGy. VMI 70 keV (A, WL/WW: 200/700 HU) exhibited consistent quantification with S, M, and L phantoms for lumen diameters greater than 6 mm. Contrast in iodine density images (B, WL/WW: 6/12 mg/mL) is easily discernible for S, M, and L phantom sizes with less conspicuous calcium shell than VMI. VNC images (C, WL/WW: 200/700 HU) have comparable quality to VMI, with increased noise at larger phantom sizes. Image quality decreased as phantom size increased.

Spectral results in the coronal plane were used to assess the influence of phantom size and lumen diameter on image quality, with findings presented in Figure 2.

### Iodine Density.

**Figure 3.**
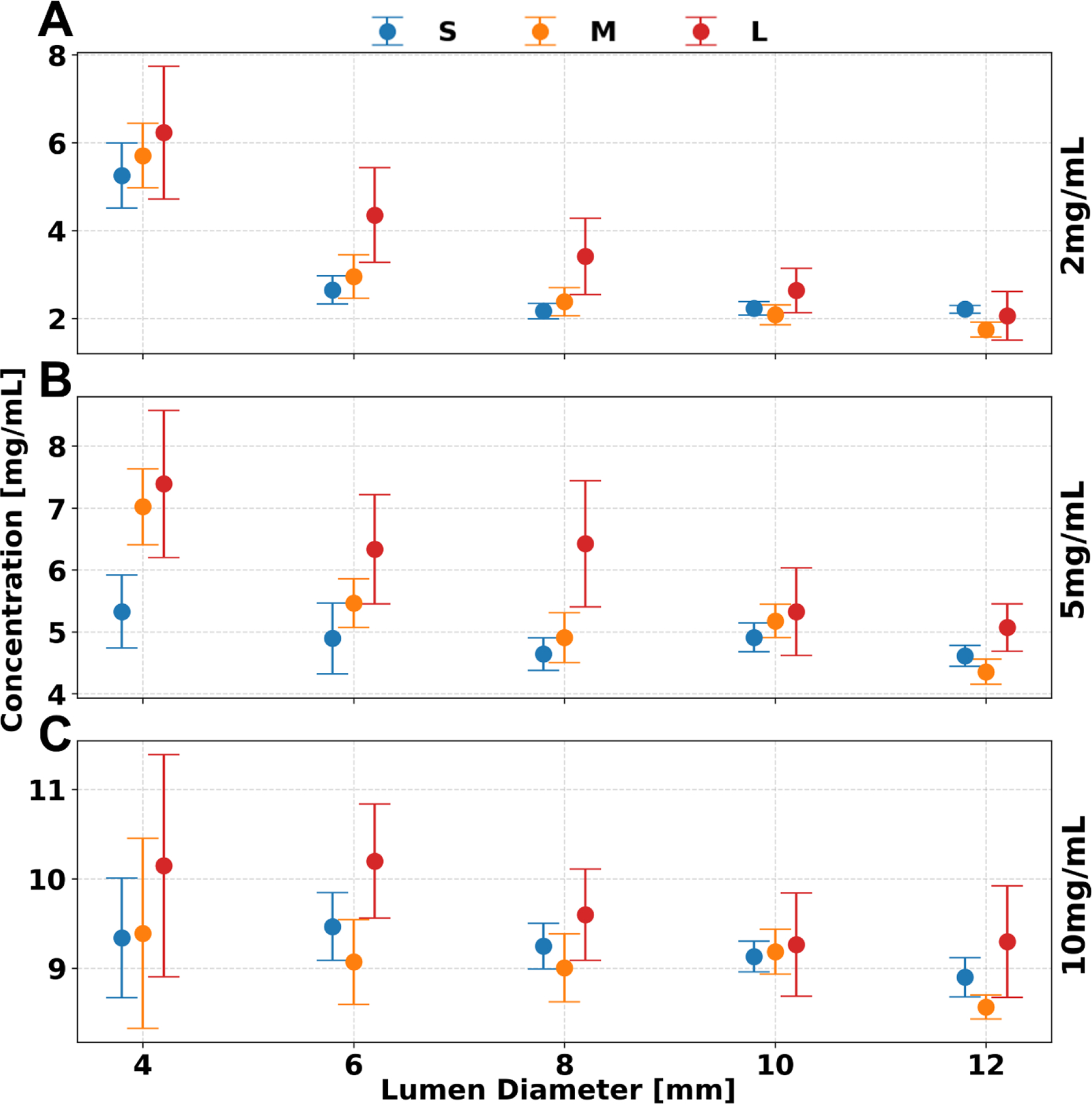
Effect of lumen diameter and phantom size on iodine quantification. Iodine material decomposition remained consistent at most phantom sizes with high accuracy at lumen diameters greater than 6 mm for iodine concentrations of 2 (**A**), 5 (**B**), and 10 (**C**) mg/mL at a radiation dose of 10 mGy. Error bars represent the standard deviation of means collected after ROI placement.

As shown in Figure 3, iodine density (ID) maps yielded more accurate results at larger lumen diameters and were affected by variations in patient size. Notably, ID showed significant dependence on lumen diameter, phantom size, and concentration (p < 1.736 ×10^−4^). ID maps had increased accuracy for lumen diameters greater than 6 mm (p < 1.736 × 10^−4^). In particular, smaller phantoms (S, M) demonstrated increased accuracy where relative differences peaked at 0.9 mg/mL across all iodine concentrations, while average difference in the M phantom remained stable at 0.1 ± 0.4, 0.4 ± 0.4, and 0.1 ± 0.2 mg/mL for concentrations of 2, 5, and 10 mg/mL, respectively. However, at a large phantom size (L), the peak difference increased to 2.1 mg/mL, with larger average differences of 0.9 ± 0.9, 1.2 ± 0.6, and 0.7 ± 0.4 mg/mL for the same concentrations. Furthermore, no significant difference was observed between the small and medium phantom (S, M) sizes whereas post-hoc analysis indicated a significant difference between each and the large (L) phantom (p < 1.736 ×10^−4^). At smaller lumen diameters (< 6 mm), quantification error increased with phantom size, while decreasing with concentration. As phantom size increased from small to large, iodine measurement differences rose—from 3.0 to 4.0 mg/mL at 2 mg/mL, 0.7 to 2.7 mg/mL at 5 mg/mL, and 0.4 to 1.2 mg/mL at 10 mg/mL concentrations. All measurement values of ID maps are recorded in Table 2.

Iodine quantification was not significantly affected by radiation dose. However, increasing the dose reduced iodine noise, resulting in improved image quality across all phantom sizes and iodine concentrations. Average quantification error remained stable between 5 and 10 mGy, with minimal changes observed in the large (L) phantom: 0.3, 0.2, and 0.1 mg/mL for iodine concentrations of 2, 5, and 10 mg/mL, respectively. Image noise on ID maps decreased with higher dose levels, dropping from 1.4 ± 0.1, 1.4 ± 0.1, and 1.3 ± 0.1 mg/mL at 5 mGy to 1.2 ± 0.1, 1.1 ± 0.0, and 1.1 ± 0.0 mg/mL at 10 mGy, across the same concentrations in the L phantom. Importantly, iodine noise was independent of iodine concentration but strongly dependent on phantom size (p < 1.736 ×10^−4^). For example, at a 5 mg/mL iodine concentration, image noise increased by 0.8 mg/mL from the small (S) to the large (L) phantom, and by 0.6 mg/mL from medium (M) to large (L) size at 10 mGy.

### VMI 70 keV.

**Figure 5.**
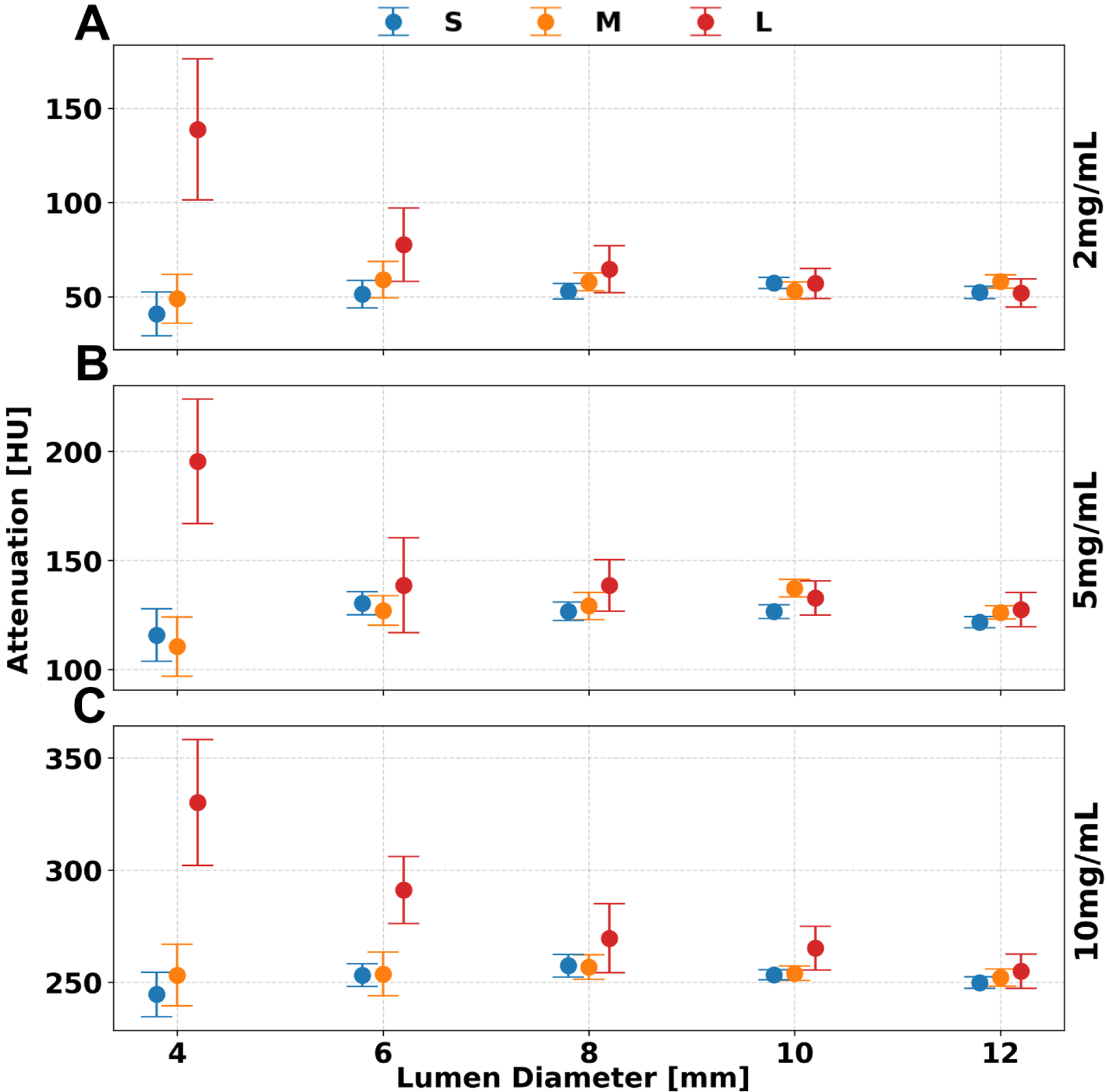
VMI 70 keV across lumen diameter, phantom size, and iodine concentration. Error in measured attenuation decreased as lumen diameter increased while phantom size increased the error within the same lumen diameter. Trends with phantom size and lumen diameter were similar for iodine concentrations of 2 (**A**), 5 (**B**), and 10 (**C**) mg/mL at 10 mGy. Error bars represent the standard deviation of values measured across all slices.

VMI 70 keV images demonstrated stable attenuation for lumen diameters greater than 6 mm, though lumen diameter, phantom size, and iodine concentration all had significant effects. While changes in lumen diameter produced only minor variations in attenuation, phantom size had a more pronounced impact on quantification accuracy (Figure 5). The average difference at larger lumen diameters did not exceed 20.3 ± 13.2 HU across all concentrations and phantom sizes, however, when only S and M phantom sizes are considered, the average error remained below 8.2 ± 4.4 HU. For smaller lumen diameters (4 mm), a clear dependency on phantom size was observed. At a radiation dose of 10 mGy, the attenuation difference in VMI 70 keV images increased markedly across phantom sizes—from −11, −5.9, and −5.2 HU in the small (S) phantom to 86.5, 73.8, and 80.3 HU in the large (L) phantom for iodine concentrations of 2, 5, and 10 mg/mL, respectively (Table 3). Radiation dose did not significantly affect the quantification accuracy of VMI 70 keV maps. However, higher dose levels resulted in a significant reduction in image noise across phantom sizes and iodine concentrations (p < 1.736 × 10^−4^).

### VNC.

**Figure 6.**
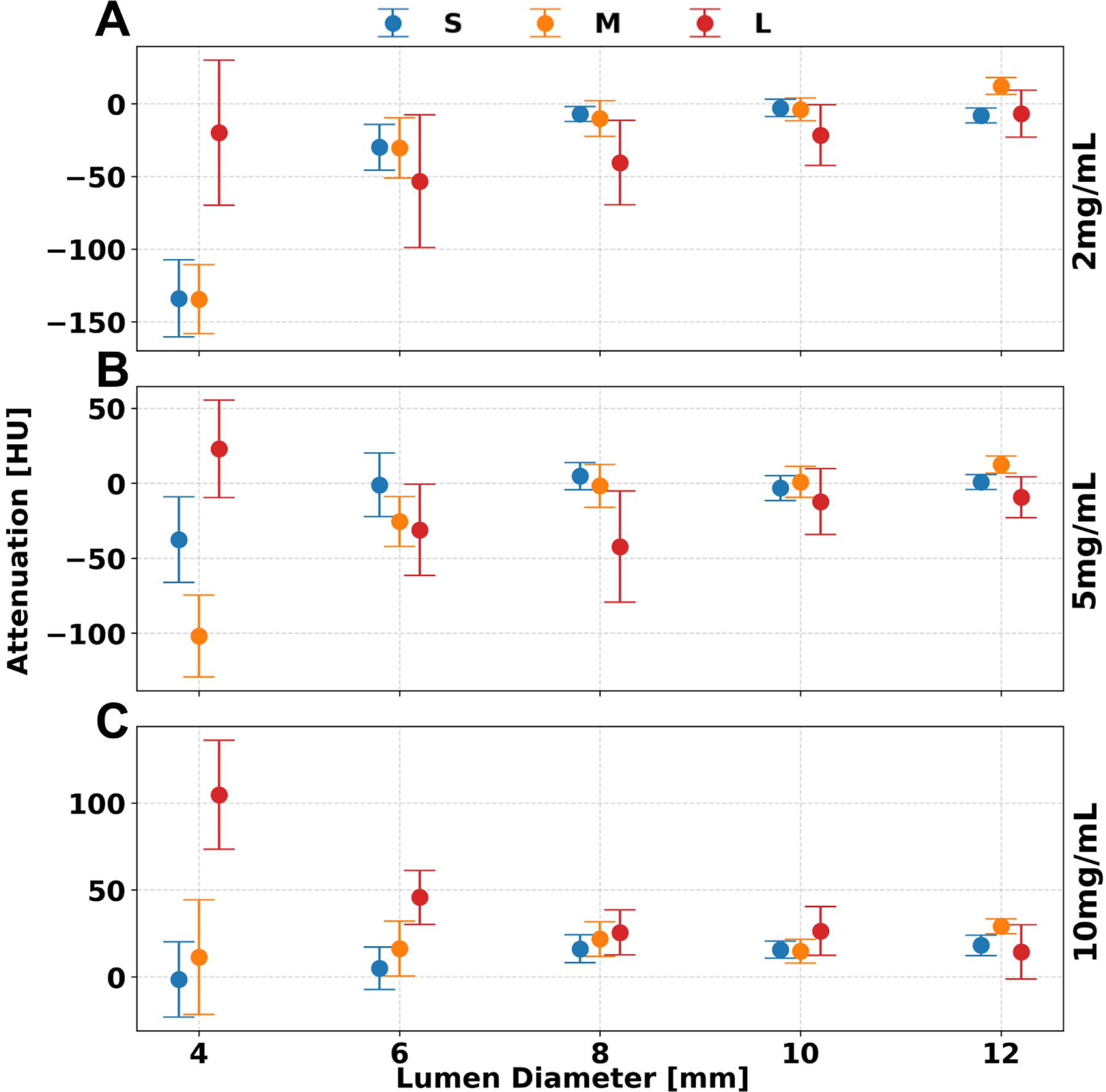
Attenuation after iodine removal across lumen diameter and phantom size. For concentrations of 2 (A), 5 (B), and 10 (C) mg/mL the replacement of iodine to water in VNC images remains consistently accurate for S, M, and L phantom sizes at a CTDI_vol_ of 10 mGy. Error bars represent standard deviation of measured mean attenuation.

Unlike previous spectral results, VNC images demonstrated consistent iodine quantification for lumen diameters greater than 8 mm, as differences between 4 mm and 6 mm diameters were not statistically significant. Iodine removal in VNC reconstructions was generally accurate across phantom sizes, with the average error—relative to the 12 mm reference value—not exceeding 24.6 ± 13.6 HU for lumen diameters ≥6 mm (Table 4), across all iodine concentrations and phantom sizes at a dose of 10 mGy (Figure 6). However, phantom size influenced iodine removal accuracy, as evidenced by an increase in error from the S to L phantom of 18.5, 24.1, and 14.3 HU for iodine concentrations of 2, 5, and 10 mg/mL, respectively. Statistical analysis confirmed significant effects of lumen diameter, phantom size, iodine concentration, and radiation dose on VNC quantification (p < 1.736 × 10^−4^).

## Discussion

Our phantom study provides a comprehensive technical assessment of a clinically available PCCT system, specifically evaluating its ability to accurately quantify iodine in the presence of calcium as well as its overall spectral performance across a diverse range of lumen diameters. The findings from this study offer insights that could inform protocol optimization and guide future research directions for CAD diagnostics and other cardiovascular conditions. Notably, our results highlight that lumen diameter and phantom size influence spectral imaging efficacy. For lumen sizes greater than 6 mm, both VMI at 70 keV and ID values demonstrated more consistent performance. However, quantification remained sensitive to phantom size and iodine concentration, while radiation dose had comparatively less impact. It is important to recognize the inherent interplay between patient size and dose, which influences imaging performance and noise characteristics.

Dual-source PCCT holds the potential to enhance cardiovascular imaging by providing superior temporal resolution, reducing motion artifacts, and enabling precise tissue characterization and quantification of materials like iodine and calcium, which are essential for accurate diagnosis and assessment of cardiovascular disease. However, factors such as lumen diameter are often overlooked in technical evaluations, despite its impact on material decomposition accuracy due to spatial resolution limitations. Prior studies examining the relationship between lumen diameter and iodine quantification in Dual-Energy CT (DECT) determined that increasing contrast concentration enhances the detectability of small lesions. Specifically, higher iodine concentrations can compensate for the limited spatial resolution needed to accurately quantify smaller lumen diameters^26^. Previously, a coronary with a similar diameter (3.5 mm) and a protruding calcification was emulated on a phantom study^27^, resulting in a bias of 0.5 mg/mL while using a high contrast concentration. In our study, iodine quantification demonstrated to be accurate across most lumen diameters (≥ 6 mm) and phantom sizes, with concentration improving contrast and quantification. Furthermore, we illustrated that higher concentrations decreased the error at smaller lumen diameters (4 mm), enhancing contrast and iodine quantification accuracy despite its close proximity to calcium. A previous characterization of ID performance identified a 0.5 mg/mL bias at an L phantom size^23^, while our average error spanned from 0.7 to 1.2 mg/mL for lumen diameters greater than 6 mm. Although the observed error was higher, the complexity introduced by smaller lumen diameters and their proximity to calcium further highlights the reliability of dual-source PCCT in iodine quantification for coronary angiography.

Coronary PCCT has the capability to always acquire spectral information when performing a scan. One of the main applications of this capability is to reconstruct VNC images from CTA studies, removing the need for a true non-contrast image, reducing the radiation dose delivered to a patient. Calcium scoring is usually performed on a non-contrast image as iodine can be confused for calcium due to their similar attenuation profiles. PCCT has been shown to have a high differentiation of calcium from surrounding tissue^19^. Furthermore, previous literature^28–30^ has explored the use of VNC images for PCCT and DECT as a replacement for non-contrast images, recognizing both difficulties—as VNC can underestimate calcification—and its potential to minimize radiation exposure. Underestimation of calcium can result in lower Agatston scores during coronary calcium scoring, potentially leading to an underrepresentation of a patient’s cardiovascular risk^28^. However, this can be corrected by a scaling factor as seen in Gassert et al^30^. Our VNC results indicate potential calcium misrepresentation in dual-source PCCT. Although iodinated contrast was effectively removed across most lumen diameters, phantom sizes, and concentrations, coronal views revealed reduced conspicuity of the calcified vessel wall. However, a new approach, denoted virtual non iodine (VNI)^28,29^ that focuses on conserving calcium on an image while removing iodine, demonstrated improved accuracy of calcium scoring compared to those from VNC, indicating the potential of spectral tools obtainable from dual-source PCCT.

This study has several limitations that should be considered when interpreting the findings. Firstly, the study was conducted using only a single PCCT system, which means that we could not assess inter-scanner variability. Differences between PCCT systems from various manufacturers could impact performance characteristics and limit the generalizability of our results across other PCCT systems. Secondly, while the PCCT system we used offers increased spatial resolution for spectral data compared to conventional CT, it currently does not simultaneously capture high spatial resolution and spectral data within a single dataset. This limitation restricts the ability to analyze fine anatomical details and spectral information together, which could impact the precision of lumen characterization in clinical practice. Additionally, the phantoms utilized in this study do not fully replicate the anatomical complexity or the diverse composition of plaques found in human patients. Realistic patient anatomy and the variability of plaque composition, which often includes a mixture of calcium, lipid, and fibrous tissue, may introduce challenges not addressed by our simplified phantom model. Moreover, while our study focused primarily on variations in iodine concentration, future research should examine a broader range of calcium concentrations and plaque types to better understand the interplay between calcium and iodine in PCCT imaging. Finally, translating these findings into clinical practice requires further studies involving patient populations. Such clinical trials are essential to confirm the diagnostic value and practical utility of PCCT for assessing cardiovascular disease in real-world settings. Nevertheless, our current study lays the technical groundwork for larger-scale studies.

In conclusion, this study provides a comprehensive technical evaluation of a clinically available PCCT system, emphasizing its ability to generate accurate spectral data for quantifying iodine in the presence of calcium across various lumen diameters and phantom sizes. Notably, we demonstrate successful iodine quantification using a dual-source PCCT approach commonly used in vascular imaging. These findings offer valuable guidance for optimizing imaging protocols in cardiovascular diagnostics, particularly for CAD, and underscore the potential of PCCT to advance non-invasive cardiovascular imaging.

## Figures and Tables

**Figure 4.**
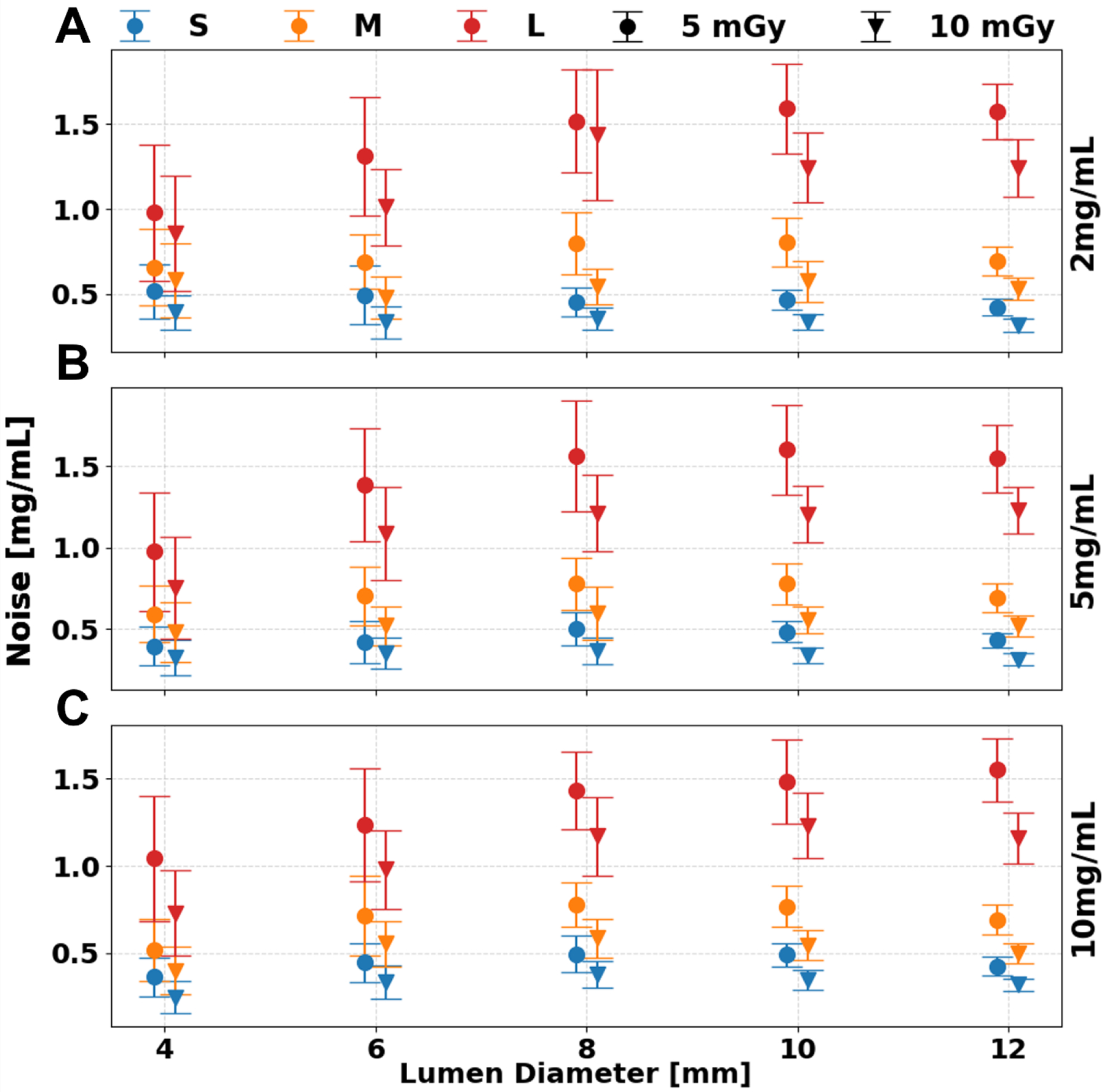
Noise of iodine density maps at a range of radiation doses and phantom sizes. Increased radiation dose mitigated the effect of larger phantom sizes for iodine concentrations of 2 (**A**), 5 (**B**), and 10 (**C**) mg/mL with error bars representing the standard deviation of ROIs collected at each insert.

**Table 1.** Acquisition and reconstruction parameters.

Parameter	
Iodine concentration [mg/mL]	2, 5, 10
CTDI_vol_ [mGy]	5, 10
Phantom size [cm]	20×20, 25×25, 30×40
Spectral results	VMI 70 keV, ID, VNC
Tube voltage [kVp]	120
Pitch	3.2
Rotation time [s]	0.25
Acquisition mode	Helical, ECG-gated
Reconstruction kernel/QIR	Bv40/3
Slice thickness/increment [mm]	0.4/0.4
Field of view [mm]	180
Matrix size	512×512

**Table 2.** Summary of measured concentrations in ID maps across lumen diameter, phantom size, and concentration at 10 mGy.

Concentration* [mg/mL]	Lumen diameter* [mm]	Measured concentration [mg/mL]			Difference [mg/mL]			Percentage Difference [%]		
		S	M	L	S	M	L	S	M	L
2	4	5.3±0.7	5.7±0.7	6.2±1.5	3.0	3.5	4.0	138.9	158.5	182.1
	6**	2.6±0.3	2.9±0.5	4.4±1.1	0.4	0.7	2.1	19.9	33.7	97.0
	8**	2.1±0.2	2.3±0.3	3.4±0.9	−0.4	0.2	1.2	−2.0	7.7	54.5
	10**	2.2±0.2	2.1±0.2	2.6±0.5	0.2	−0.1	0.4	1.0	−5.8	19.3
	12**	2.2±0.1	1.7±0.2	2.1±0.6	-	−0.5	−0.1	-	−21.1	−6.8
5	4	5.3±0.6	7.0±0.6	7.4±1.2	0.7	2.4	2.7	15.5	52.2	60.2
	6**	4.9±0.6	5.5±0.4	6.3±0.8	0.3	0.9	1.7	6.1	18.5	37.3
	8**	4.6±0.3	4.9±0.4	6.4±1.0	0.0	0.3	1.8	0.6	6.4	39.2
	10**	4.9±0.2	5.2±0.3	5.3±0.7	0.3	0.6	0.7	6.5	12.2	15.4
	12**	4.6±0.1	4.4±0.2	5.1±0.4	-	−0.3	0.5	-	−5.6	9.9
10	4	9.3±0.7	9.3±1.1	10.1±1.2	0.4	0.5	1.2	5.0	5.5	14.4
	6**	9.5±0.4	9.1±0.5	10.2±0.6	0.6	0.2	1.3	6.4	1.9	14.6
	8**	9.3±0.3	9.0±0.4	9.6±0.5	0.4	0.1	0.7	3.9	1.2	7.9
	10**	9.1±0.2	9.1±0.3	9.3±0.6	0.2	0.3	0.4	2.6	3.2	4.1
	12**	8.9±0.2	8.6±0.1	9.3±0.6	-	−0.3	0.4	-	−3.7	4.5

Variables marked with (*) are those were a significant difference between parameters was estimated using Kruskal-Wallis test. All parameters marked with (**) are those with a significant difference to the 4 mm lumen diameter of their respective concentration according to a Dunn post-hoc analysis.

**Table 3.** Measured attenuation of VMI 70 keV images at a range of concentrations and phantom sizes.

Concentration* [mg/mL]	Lumen Diameter* [mm]	Measured Attenuation [HU]			Difference [HU]		
		S	M	L	S	M	L
2	4	40.9±11.7	48.9±12.9	138.8±37.4	−11.4	−3.4	86.5
	6**	51.4±7.3	49.3±9.7	77.6±19.5	−0.9	6.8	25.2
	8**	53.0±4.2	57.8±4.7	64.6±12.4	0.7	5.5	12.2
	10**	57.3±2.9	53.2±4.6	57.0±8.0	5.0	0.9	4.7
	12**	52.3±3.3	58.1±3.6	52.0±7.5	-	5.8	−0.3
5	4	115.7±12.0	110.5±13.6	195.4±28.5	−5.9	−11.1	73.8
	6**	130.3±5.3	127.0±6.7	138.6±21.7	8.7	5.4	17.0
	8**	126.7±4.2	129.1±6.2	138.5±11.8	5.0	7.5	16.9
	10**	126.5±3.2	137.3±4.0	132.8±7.9	4.9	15.6	11.1
	12**	121.7±2.6	126.1±3.0	127.5±7.8	-	4.5	5.8
10	4	244.6±9.9	253.2±13.7	330.1±28.0	−5.2	3.3	80.3
	6**	253.2±5.1	253.7±9.8	291.1±14.9	3.3	3.8	41.2
	8**	257.4±5.0	256.8±5.4	269.6±15.3	7.5	6.9	19.7
	10**	253.4±2.3	254.0±3.3	265.2±9.7	3.5	4.2	15.3
	12**	249.9±2.6	252.1±3.9	255.0±7.6	-	2.3	5.1

Variables marked with (*) are those were a significant difference between parameters was estimated using Kruskal-Wallis test. All parameters marked with (**) are those with a significant difference to the 4 mm lumen diameter of their respective concentration according to a Dunn post-hoc analysis.

**Table 4.** Summary of measured values of VNC images according to concentration, lumen diameter, and phantom size.

Concentration* [mg/mL]	Lumen Diameter* [mm]	Measured attenuation [HU]			Difference [HU]		
		S	M	L	S	M	L
2	4	−134.0±26.5	−134.5±23.6	−19.9±49.9	−125.8	−126.4	−11.7
	6	−30.0±15.8	−30.3±20.7	−53.4±45.7	−21.8	−22.2	−45.3
	8**	−7.2±5.1	−10.2±12.2	−40.5±29.0	1.0	−2.0	−32.4
	10**	−2.9±6.0	−3.9±7.8	−21.6±20.8	5.3	4.2	−13.4
	12**	−8.2±5.1	12.2±5.7	−6.8±16.1	-	20.4	1.4
5	4	−37.6±28.5	−101.9±27.2	22.9±32.5	−38.4	−102.7	22.1
	6	−1.1±21.1	−25.4±16.6	−31.1±30.4	−1.9	−26.2	−31.9
	8**	4.7±9.1	−1.7±14.4	−42.2±37.1	3.9	−2.6	−43.1
	10**	−3.2±8.3	0.9±10.2	−12.3±21.9	−4.0	0.1	−13.1
	12**	0.8±4.9	12.5±5.7	−9.3±13.6	-	11.7	−10.1
10	4	−1.5±21.5	11.4±33.0	104.7±31.4	−19.6	−6.7	86.6
	6	5.0±12.1	16.4±15.8	45.8±15.5	−13.1	−1.7	27.7
	8**	16.1±8.0	21.8±9.9	25.5±12.9	−2.0	3.7	7.4
	10**	15.6±4.9	14.8±6.9	26.4±14.0	−2.5	−3.3	8.3
	12**	18.1±5.8	29.1±4.2	14.3±15.6	-	11.0	−3.8

Variables marked with (*) are those were a significant difference between parameters was estimated using Kruskal-Wallis test. All parameters marked with (**) are those with a significant difference to the 4 mm lumen diameter of their respective concentration according to a Dunn post-hoc analysis.
